# Knowledge about complementary, alternative and integrative medicine (CAM) among registered health care providers in Swedish surgical care: a national survey among university hospitals

**DOI:** 10.1186/1472-6882-12-42

**Published:** 2012-04-12

**Authors:** Kristofer Bjerså, Elisabet Stener Victorin, Monika Fagevik Olsén

**Affiliations:** 1 Department of Surgery, Institute of Clinical Sciences, Sahlgrenska Academy, University of Gothenburg, Gothenburg, Sweden; 2 Department of Surgery, Sahlgrenska University Hospital, Gothenburg 41345, Sweden; 3 Institute of Neuroscience and Physiology, Sahlgrenska Academy, University of Gothenburg, Gothenburg, Sweden

**Keywords:** Complementary therapies, CAM, Sweden, Surgery, Knowledge

## Abstract

**Background:**

Previous studies show an increased interest and usage of complementary and alternative medicine (CAM) in the general population and among health care workers both internationally and nationally. CAM usage is also reported to be common among surgical patients. Earlier international studies have reported that a large amount of surgical patients use it prior to and after surgery. Recent publications indicate a weak knowledge about CAM among health care workers. However the current situation in Sweden is unknown. The aim of this study was therefore to explore perceived knowledge about CAM among registered healthcare professions in surgical departments at Swedish university hospitals.

**Method:**

A questionnaire was distributed to 1757 registered physicians, nurses and physiotherapists in surgical wards at the seven university hospitals in Sweden from spring 2010 to spring 2011. The questionnaire included classification of 21 therapies into conventional, complementary, alternative and integrative, and whether patients were recommended these therapies. Questions concerning knowledge, research, and patient communication about CAM were also included.

**Result:**

A total of 737 (42.0%) questionnaires were returned. Therapies classified as complementary; were massage, manual therapies, yoga and acupuncture. Alternative therapies; were herbal medicine, dietary supplements, homeopathy and healing. Classification to integrative therapy was low, and unfamiliar therapies were Bowen therapy, iridology and Rosen method. Therapies recommended by > 40% off the participants were massage and acupuncture. Knowledge and research about CAM was valued as minor or none at all by 95.7% respectively 99.2%. Importance of possessing knowledge about it was valued as important by 80.9%. It was believed by 61.2% that more research funding should be addressed to CAM research, 72.8% were interested in reading CAM-research results, and 27.8% would consider taking part in such research. Half of the participants (55.8%) were positive to learning such therapy. Communication about CAM between patients and the health care professions was found to be rare.

**Conclusion:**

There is a lack of knowledge about CAM and research about it among registered health care professions in Swedish surgical care. However, in contrast to previous studies the results revealed that the majority perceived it as important to gain knowledge in this field.

## Background

The increased usage of complementary and alternative medicine (CAM) in Europe and America during the last decades is well acknowledged in research literature [1-3]. This growing trend has also been seen in the population of the Scandinavian countries during the last decades [4,5]. Typical CAM users in the Scandinavian countries are people with higher education, lower self-perceived health, and women. There are however differences between the countries [4]. In a Swedish population (Stockholm county), the most commonly used therapies found were massage, natural remedies and chiropractic. In Norway however, homeopathy, chiropractic and acupuncture were most common, and reflexology, massage and homeopathy in Denmark [4].

An elevating interest about CAM has also been observed among health care professions. Their attitudes and usage have been reported both internationally [6-13] and in Scandinavian countries [14-16]. Consensus of these previous studies shows a gap in current knowledge about CAM and the wish for such knowledge. The reason for this increasing interest in CAM in Norway and Denmark is discussed as the growing body of evidence about CAM and the personal interest among the employees [14]. International comparisons of CAM usage with Scandinavian countries can be made, but differences in culture and health care service may influence the perceptions and should therefore be taken into consideration. This may also influence the health care providers' attitude toward CAM [17]. There are also differences between the Scandinavian countries in their policy toward CAM [16,18].

Results from studies among surgical patients in North America indicate a high usage of CAM, and a significant number of patients consider using it during the perioperative phase [19-23]. It is not known to what extent this applies for Swedish surgical patients, but the increased usage in the general population may however affect the health care providers' perception. A recent qualitative study among registered Swedish healthcare providers in surgical care indicates a need for policies on management, education and research in CAM in Sweden [16]. It is important to test and verify these results in a larger national study.

Thus, the aim of this study was to explore perceived knowledge about CAM among registered healthcare professions in surgical departments at Swedish university hospitals.

## Methods

This study was conducted as a cross-sectional questionnaire study among all seven Swedish university hospitals.

### Definitions

In this paper the definitions of the concepts (conventional, complementary, alternative and integrative) are adjusted to the Swedish healthcare system and presented in Table 1. The definitions are based on the definitions given by The National Centre for Complementary and Alternative Medicine [24]. Use of the term "medicine" and "therapy/-ies" is considers equal based on the MESH-term "complementary therapies".

**Table 1:** Definitions of conventional, complementary, alternative and integrative medicine/therapy presented in this study

Conventional medicine/therapy	Care given by public hospitals, district health care centres or home nursing
Alternative medicine/therapy	Treatments given instead of conventional medicine/therapies

Complementary medicine/therapy	Treatments given parallel with conventional medicine/therapies, but without dialogue between the two caregivers

Integrative medicine/therapy	Treatments given in collaboration and dialog between conventional medicine/therapies and alternative- and complementary medicine/therapies

### Study design

A questionnaire was created with inspiration from the CAM Health Belief Questionnaire (CHBQ), a Norwegian attitude of CAM study among oncology profession, the International Questionnaire to measure use of Complementary and Alternative medicine (I-CAM-Q) and the result from our previous qualitative Swedish study [15,16,25-27]. The questionnaire was initially tested on 17 nurses and five physicians in surgical departments at two different hospitals. The questionnaire was remodelled from their result and comments. A second test was preformed among 21 other nurses and four physicians at the same surgical departments initially used. Just minor adjustments were made from that result. The final and distributed questionnaire was five pages and consisted of:

• A front page including information and definitions of the area.

• A list of 23 therapies which were to be classified into conventional, complementary, alternative, integrative or unknown therapy, and also to indicate whether they would recommend the therapy to family or/and to patients.

• A total of 11 questions studying knowledge, research and, dialog with patients about CAM.

• Twelve questions based on a translated version of CHBQ into Swedish. Modification was made to response scale from seven to six point response scale, and exclusion of question two. Additional questions regarding spirituality, cost of treatment and guidelines were included. Results from this part of the questionnaire are not reported in this paper.

• Personal usage of CAM therapies, total cost for that treatment and experienced effect (not reported in this paper).

• Demographical data; profession, surgical speciality, level of experience in profession, experience in surgical care, gender, year of birth. Also personal contact/use with conventional healthcare, education in and performance of CAM (also not reported in this paper).

### Study sample and data collection

Between March 2010 and April 2011 contact was made with 71 surgical wards at the seven university hospitals in Sweden. These wards included 10 different surgical specialities; upper and lower gastrointestinal, urology, plastic/reconstructive, cardiothoracic, emergency and trauma, mammae, endocrinological, and vascular. The heads of departments', matrons, and other head of staff approved the distribution of the questionnaire to the targeted professions; physicians, nutritionists, nurses, physiotherapists. Fifty nine of the 71 wards participated with one to all four professions. A total of 1776 paper questionnaires were distributed to the participants' workplace post-boxes with a returning envelope. For practical reasons, 63 questionnaires were distributed to home addresses. Reminders were sent two and four weeks after initial distribution.

A decision was made to exclude dieticians based on the low population size (n = 19) with the risk of compromising personal integrity.

A non-response analysis was conducted for control on distribution among professions and of gender in the population at the surgical wards in university hospitals. Data were retrieved from each of the hospitals Department of Human Resource.

### Data analysis

Demography was compiled using Microsoft Excel and presented in numbers (n) and percentage (%). Analyses were performed using IBM SPSS version 19.0. Chi-2 test (Goodness-of-fit, Pearson) was used for comparison of nominal variables; gender and wish for knowledge. Correlation between variables with ordinal data; patient asking and participants asking, was conducted using Spearman correlation coefficient. One sample *t*-test was used for comparison of age between the population and the participants in the non-response analysis. Kruskal-Wallis with Mann-Whitney-U post hoc and Bonferroni correction were used for ordinal data in more than two groups; profession and knowledge. Significant levels were set to p < 0.05, and correlation levels (r_s_) in this study were defined as: weak correlation 0.3-0.5 and strong correlation > 0.6.

### Ethical consideration

Approval for this study was given by The Regional Ethical Review Board in Gothenburg, Sweden (Dnr.066-09). Approval was acquired from all heads/directors of departments, matrons, and other heads of staff. The front page of the questionnaire stated that participation was voluntary, data would be handled confidentially, and that the results would be presented at group level.

## Result

A total of 1757 questionnaires were posted and 737 (42.0%) were returned. Range of response among the seven university hospitals was 33.2% to 52.1%. Demographical data is reported in Table 2. The participants were on average 40.3 years of age, 77.5% (n_total _= 714) were women, with nurses comprising the most dominant profession (70.4%; n_total _= 737). Work life experience in the profession, as well as in surgical care, varied between the professions.

**Table 2:** Participants demography

		Physician	Nurses	Physiotherapists	Total
**Study sample (Participants)**					

Total (Distributed)		536	1140	81	1757

Returned		158	519	60	737

Answering frequency		29.5%	45.5%	74.1%	42.0%

Age	Mean (SD)	47.8 (11.3)	37.9 (10.3)	41.6 (10.1)	40.3 (11.2)
	
	Min-Max	27-70	23-68	28-63	23-70

Gender Male/Female		74.7%/25.3% (112/38)	8.3%/91.7% (42/462)	11.9%/88.1% (7/52)	22.5%/77.5% (161/553)

Working life experience in the profession:	0-2 y	4.0%	25.4%	7.0%	19.4%
	
	3-5 y	13.3%	20.2%	21.1%	18.8%
	
	6-10 y	17.9%	19.2%	21.1%	19.1%
	
	11-20 y	28.1%	18.8%	29.8%	21.9%
	
	> 20 y	35.8%	16.4%	21.1%	20.9%

Working life experience in surgical care:	0-2 y	7.3%	28.5%	18.6%	23.3%
	
	3-5 y	11.9%	20.7%	30.5%	19.6%
	
	6-10 y	22.5%	19.9%	20.3%	20.5%
	
	11-20 y	22.5%	15.8%	18.6%	17.4%
	
	> 20 y	35.8%	15.2%	11.9%	19.2%

**Population data**					

Age Mean		46.8 (n = 703)	38.0 (n = 1764)		40.5 (n = 2467)

Gender Male/Female		73.0%/27.0% (n = 760)	8.4%/91.6% (n = 1704)		28.3%/71.7% (n = 2464)

A non-response analysis was conducted for comparisons between the participants in the study toward the population from the targeted surgical departments. It was only possible to retrieve reliable data for physicians and nurses as shown in Table 2. There were no statistical differences in gender between physicians compared with the population of physicians at the surgical wards in the university hospitals (p = 0.646). Likewise for the nurses (p = 0.984). Corresponding figures regarding age were p = 0.263 and p = 0.805. Differences were found in distribution between physicians and nurses, with significantly fewer physicians than nurses compared with the population (p < 0.001).

### Therapy classification and recommendation

Classification of the 21 therapies or areas of care were made into; conventional, complementary, alternative, integrative medicine, or therapy unknown, and are given in Table 3. The therapies classified most frequently as complementary (> 40%) were; massage, manual therapies (chiropractic, naprapathy, osteopathy), yoga and acupuncture. The most frequently classified alternative therapies (> 60%) included; herbal medicine and dietary supplements, homeopathy and healing. The proportion of therapies classified into integrative therapy was low, with only acupuncture and psychotherapy having a frequency over 25%. The most unfamiliar therapies (> 70%) included; Bowen therapy, iridology, and the Rosen method.

**Table 3:** Classification of therapeutical areas and the frequency of recommendation.

	Conventional therapy	Complementary therapy	Alternative therapy	Integrative therapy	Therapy unknown	Would recommend it to patients
**Ayurveda **(n = 700)	0% (n = 0)	5.9% (n = 41)	24.9% (n = 174)	0.9% (n = 6)	68.4% (n = 479)	2.6% (n = 18)

**Homeopathy **(n = 694)	0.4% n = 3)	8.7% (n = 60)	66.6% (n = 462)	2.0% n = 14)	22.3% (n = 155)	4.0% (n = 28)

**Psychotherapy, CBT **(n = 693)	46.0% (n = 319)	20.2% (n = 140)	3.8% (n = 26)	26.3% (n = 182)	3.8% (n = 26)	59.3 (n = 411)

**Meditation, Mindfullness, etc**. (n = 695)	1.6% (n = 11)	39.0% (n = 271)	40.7% (n = 283)	8.1% (n = 56)	10.6% (n = 74)	27.2 (n = 189)

**Healing, Reiki, etc**. (n = 697)	0% (n = 0)	11.0% (n = 77)	65.7% (n = 458)	1.3% (n = 9)	22.0% (n = 153)	3.2% (n = 22)

**Yoga **(n = 693)	0.7% (n = 5)	42.4% (n = 294)	43.7% (n = 303)	8.2% (n = 57)	4.9% (n = 34)	33.3% (n = 231)

**Nursing **(n = 697)	81.6% (n = 569)	3.7% (n = 26)	0.4% (n = 3)	12.5% (n = 87)	1.7% (n = 12)	61.3% (n = 428)

**T'ai chi, Qi gong **(n = 701)	0.6% (n = 4)	34.1% (n = 239)	44.9% (n = 315)	5.6% (n = 39)	14.8% (n = 104)	21.5% (n = 151)

**Acupuncture, Acupressure** (n = 686)	14.1% (n = 97)	40.5% (n = 278)	16.8% (n = 115)	27.1% (n = 186)	1.5% (n = 10)	47.6% (n = 327)

**Orthopaedic manual therapy (OMT/OMI) **(n = 701)	20.3% (n = 142)	13.4% (n = 87)	3.9% (n = 27)	8.1% (n = 57)	55.3% n = 388)	20.1% (n = 141)

**Massage, shiatsu, tactile massage, etc**. (n = 687)	8.3% n = 57)	47.2% (n = 324)	19.4% (n = 133)	20.5% (n = 141)	4.7% (n = 32)	49.9% (n = 343)

**Chiropractic, Naprapathy, Osteopathy **(n = 688)	10.3% (n = 71)	44.8% (n = 308)	26.2% (n = 180)	16.1% (n = 111)	2.6% (n = 18)	38.1% (n = 263)

**Physiotherapy **(n = 694)	67.1% (n = 466)	7.8% (n = 54)	1.9% (n = 13)	22.6% (n = 157)	0.6% (n = 4)	66.7% (n = 463)

**Herbal medicine, Dietary supplement **(n = 700)	0.7% (n = 5)	20.6% (n = 144)	66.9% (n = 468)	5.0% (n = 35)	6.9% (n = 48)	13.7% (n = 96)

**Bowen therapy **(n = 706)	0% (n = 0)	0.3% (n = 2)	4.1% (n = 29)	0.3% (n = 2)	95.3% (n = 673)	0% (n = 0)

**Iridology **(n = 710)	1.1% (n = 8)	1.4% (n = 10)	21.1% (n = 150)	0.8% (n = 6)	75.5% (n = 536)	1.3% (n = 9)

**Occupational therapy** (n = 702)	67.4% (n = 473)	8.0% (n = 56)	2.0% (n = 14)	20.4% (n = 144)	2.1% (n = 15)	61.5% (n = 432)

**Kinesiology **(n = 703)	1.7% (n = 12)	5.1% (n = 36)	23.5% (n = 165)	1.0% (n = 7)	68.7% (n = 483)	3.8% (n = 27)

**Sense therapies (e.g. light-, music-, aroma therapy)** (n = 702)	4.4% (n = 31)	29.6% (n = 208)	32.8% (n = 230)	15.1% (n = 106)	18.1% (n = 127)	21.4% (n = 150)

**Rosen method **(n = 707)	0.4% (n = 3)	5.2% (n = 37)	22.5% (n = 159)	1.0% (n = 7)	70.9 (n = 501)	2.4% (n = 17)

**Reflexology/zone therapy** (n = 700)	0.3% (n = 2)	16.1% (n = 113)	47.3% (n = 331)	3.9% (n = 27)	32.4% (n = 227)	8.1% (n = 57)

n = response rate (number of answers)

Table 3 also includes whether participants would recommend the therapies to patients. Apart from the conventional therapies included in the questionnaire (Psychotherapy, Nursing, Physiotherapy, Occupational therapy), the most commonly recommended therapies (> 40%) included massage and acupuncture/acupressure. There was a significant difference between the professions in total recommendation (p < 0.001) with physiotherapists making significantly more recommendations in comparison to physicians and nurses. There was no difference between physicians and nurses.

### Knowledge about CAM

**Figure 1:**
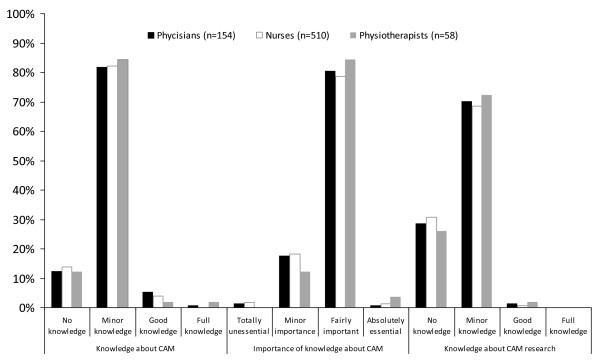
**Knowledge about CAM, importance of knowledge, and knowledge about CAM-research**.

Perceived knowledge about CAM vas valued by 95.7% (n_total _= 723) as minor or no knowledge at all (Figure 1), with no statistical difference between professions. Though, 80.9% (n_total _= 721) valued the significance of possessing knowledge about CAM as important (Figure 1), with a significant difference between professions. Significantly fewer nurses than physicians and physiotherapists perceived it as less important (p = 0.008), but there was no difference between physicians and physiotherapists. Of the total number of participants, 68.7% (n_total _= 716), wished for more knowledge about CAM, with no statistical difference between professions (p = 0.256).

Education in any CAM therapy was reported by 8.5% (n_total _= 708) of the participants, with a significant difference between the professions. Significantly more physiotherapists (34.5%) were educated in some CAM therapy (p < 0.001) in comparison to physicians and nurses. There was no significant difference between physicians and nurses. The use of any CAM therapy in clinical practice was reported by 1.8% of the participants, and 4.9% used it during free time (n_total _= 720). Physiotherapists used it significantly more often in clinical practice (10.2%; p < 0.001) compared to physicians and nurses. There was no significant difference between physicians and nurses. There was no significant difference between the professions in practice of CAM therapy during free time.

Just over half of the participants, (55.8%;n_total _= 708), were positive to learning a therapy in the CAM field, with a statistical difference between professions (p = 0.016). Physicians were significantly more interested in learning a therapy in comparison to nurses and physiotherapists (64.9%;n_total _= 151). There were no significant differences between nurses and physiotherapists. Those willing to learn a therapy graded their knowledge about CAM higher (p < 0.001) than those showing less interest.

Knowledge about research in the field of CAM was rated by 99.2% (n_total _= 722), as minor or never heard of (Figure 1). Though, 72.8% (n_total _= 721) of the participants were positive to take note of such results. A total of 61.2%, (n_total _= 699) believed that more research funding should be reserved for CAM research, and 27.8% (n_total _= 719) would consider taking part in such projects. There was no difference between professions in the result of questions concerning CAM research.

### Patient communication with regard to CAM

**Figure 2:**
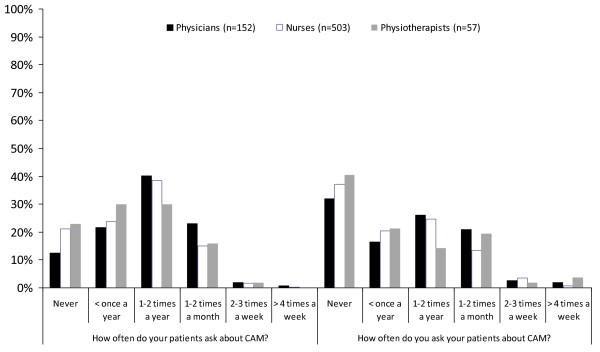
**Patient-Health care profession dialog of CAM**.

According to the results of the questionnaires, patients rarely brought up issues concerning CAM with the participants, and very seldom did the participants discuss it with their patients (Figure 2). A weak correlation was found between those participants who perceived that their patients asked more frequently and those participants who more often asked their patients regarding CAM usage (r = 0.557;p < 0.001). There were no differences between professions when asking patients about CAM. However, the perception of patients asking differed between professions (p = 0.005), where physicians were asked significantly more frequently by patients compared to nurses and physiotherapists. There were no difference between nurses and physiotherapists.

## Discussion

The main findings in this paper of Swedish healthcare professionals in surgical care shows perceived classifications of CAM therapies, their lack of knowledge in CAM and CAM research, and their low level of communication regarding CAM usage with their patients.

The classification of the assigned therapies emerged into definitions of the four domains (conventional, complementary, alternative, and integrative) as follows;

Conventional therapy included treatment given by healthcare disciplines working in public health (nursing, physiotherapy, occupational and psychotherapy). Complementary therapies included those accepted for use in the public health system (acupuncture, acupressure, massage, chiropractic, etc.). Alternative therapies were those not accepted for use in the public health system (homeopathy, healing forms, reflexology, and herbal medicine). This classification can be compared to Risberg et al.'s [15] conclusion where they found that the term "Alternative therapy" was perceived as much more negative then the term "Complementary therapy" by healthcare professions in the field of oncology in Norway.

Surprisingly, no therapy was clearly classified as integrative by the participants. This might be due to the wide interpretation of the term [28], and the recent definition of the term in Swedish literature [29] as well as the introduction of the MeSH term in 2009.

The results of this study in comparison to a German study show that CAM is less frequently recommended to patients in Sweden than in Germany [7]. However, German and Swedish healthcare systems, cultures and attitudes towards CAM might not be comparable.

Massage and acupuncture/acupressure were the therapies most commonly referred to in this study. Interestingly, Berman et al. [30] report almost identical results in referral of patients to different CAM therapies by American rheumatologists in the beginning of the 21st century. It is also notable that previous studies have found that rural healthcare providers are more likely to recommend it to their patients in comparison with their urban colleagues [31]. The results of our study, where the professions worked in surgical wards at university hospitals, may therefore be interpreted from this perspective.

The present study displays differences to a previous report among Norwegian oncology professionals [15]. The oncology study classified a greater number of therapies as complementary in contrast to the present study, where classification into alternative was much higher for the comparative therapies. Also the classification of "unknown therapy" was higher in this study compared with the Norwegian, with exception of Ayurveda (68% versus 73%). This may be due to a difference in perceptions of CAM between professions in surgical care and oncology, or/and between Sweden and Norway.

Some therapies were obviously difficult to sort into the complementary or alternative domain (meditation forms, yoga, tai chi, qi gong, sense therapies). This might be explained as therapies being in transition of perceived definition. Some therapies have been tested and used in public health during the last decades and moved from alternative to complementary e.g. acupuncture and manual therapies. Therapies that are in transition from alternative towards complementary become diffuse in classification. Yoga and meditation are good examples of such therapies, which have been tested in public health and used in health centres, and thereby gained more acceptances.

Lack of knowledge among registered Swedish healthcare professions in surgical care as shown in this study, has been reported in a previous qualitative study [16]. Similar findings of lack of knowledge among healthcare workers have also been reported internationally [9,12,32,33]. In contrast, 60% of Italian nurses claim, in a questionnaire study, to have knowledge about CAM [8].

Bjerså et al. [16], as well as Hirschkorn and Bourgeault [34], found that obstacles to retrieve the knowledge were lack of time and a perceived difficulty to access CAM knowledge and research results. The results of the present study showed that registered healthcare workers felt that possessing knowledge regarding CAM was of average importance. This is also supported in other international publications that healthcare workers want to learn more about CAM [6,13].

In this paper, as well as in the previous study by our research group [16], knowledge about CAM research was very low or non existent. The previous study also showed that conceptions in the result to be contradictive. Despite the low knowledge level, CAM research was criticised for being of low quality with many biases. It was also perceived that it was vital to create evidential research in the process for judging whether to use therapies or not. This may explain why over 60% of the participants in this study thought that more resources should be addressed to CAM research. The conclusion of a national review of CAM states that more research should be addressed to measure current consumption, effect, risks and adverse effects, and the economical dimensions in CAM usage [35].

Communication between patients and the caregiver regarding CAM was perceived as rare in this study as well as in previous international publications [6,9,11,13]. Maybe lack of knowledge discourages the caregiver from bringing up the subject and having to face questions they are not capable of answering, which is supported by a previous study among paediatricians [36]. A suggestion on how to approach this problem as a clinician is by using the communication recommendation developed by Schofield et al. [37].

It is also important to put the results from this study in a national perspective. In the late seventies, Jacobsson [38] found that 22% of a random sample of Swedish physicians asked their patients frequent or sometimes about their use of CAM. He also found that 56% of the physicians believed that patients rarely told their physician about CAM usage. Now, 30 years later, that rate still remains as shown in Figure 2. In a questionnaire study among Swedish physicians in the early 1990's, Lynöe and Svensson [39] asked for attitudes toward different therapies in the field of CAM. Complementary therapies in this study were acupuncture, homeopathy, manual therapies, reflexology and natural remedies. Comparisons of the percentage of "unknown therapies" with the present study shows that only acupuncture was less known by the professions in surgical care and the physicians in this study. Why this has not been affected by the increased usage of CAM in the general Swedish population is unclear. Jacobsson [38] reports that 52% of the physicians did not find it valuable to gain further knowledge in the area. This view has however changed drastically as shown in Figure 1.

There were significant differences between the professions in this study. Physicians were generally more interested in learning about CAM therapies and were most frequently asked about it by patients. Nurses regarded it as less important to have knowledge about CAM in comparison to the other professions. Physiotherapists were, as a group, more educated in CAM therapies and used it in more often their professional practice. They also made most recommendations to patients in comparison to the other professions. Due to the low answering frequency, it is difficult to generalise these results to the population of professionals working in Swedish surgical care. As Hirschkorn and Bourgeault [34] points out; there is no simple conclusion to draw in differences between healthcare professions in their thoughts about CAM due to the extensive numbers of both personal, professional and organisational affecting variables.

According to Wang et al. [19,20] and Norred [22] the majority of surgically treated patients use CAM, including prayer. A more recent study shows a general CAM usage of approximately 27% among surgical patients [23]. How this distribution correlates with Swedish patients is not yet studied. It is however concluded that the use of CAM in the Swedish general population has increased during the last decades. It is thus important to give attention to the patients' usage, knowledge and attitude towards CAM in future research.

### Methodological limitations

This study has several methodological limitations. There is always a risk choosing a questionnaire survey as a method for measuring. One risk is the reliability and validity of the questionnaire. The questions in this study were created from the results of previous studies [15,16,25-27]. The purpose has not been to create a new questionnaire, but to use previous knowledge and adjust it to the present aim. Also, the questionnaire was tested and adjusted in the present context twice before distribution, which justifies its usability.

Willson et al. [40] call attention to two factors to errors in response. Definition of terms used in the survey is the first factor. It is a risk that the researcher's definition of the terms does not correlate with the participants. This could be managed by including definitions in the survey, which has been made in this study. The other factor is the notion of self-concept. This implies to the participants' own view of themselves in relation to the term. For example, a participant view of how they are and what they should be doing does not correspond with the true fact. This error is hard to account for and minimize. Also the fact that some therapies in this study are merged into concepts (e.g. herbal medicine, natural remedies and nutritional supplements) could affect the participants' response and the study result.

Another risk is low response rate. In this study, 42.0% of the questionnaires were answered and returned. Similar, international studies have reported a response rate of between 18% and 61% [7,10,11,15,30,36,41,42], which make this study comparable. Hence, it is of importance to be aware of differences in health care systems, organisations, or responsibilities and characters in the different professions when comparing the content of this result with other international studies. The rather extensive questionnaire of five pages may also have contributed to a low response rate.

Just another risk is that the participants in this study could be more emotionally reactive to the subject CAM than those who did not participate. It is therefore important to be aware that there may be differences between the participants and the target population. It is not possible, from these results, to draw any general conclusions. Thus, these results confirm findings from our previous qualitative study [16] and puts in into a national perspective. The result should be regarded as a first insight into Swedish registered healthcare professions approach towards CAM.

## Conclusion

There is a lack of knowledge about CAM and CAM research among registered healthcare professionals in Swedish surgical care. These results both resemble and differ from other international studies, as does the classification of different therapies. The participants did however perceive it important to gain knowledge about CAM, which is in contrast to previous national studies performed over the last 30 years.

## Competing interests

The authors declare that they have no competing interests, and were free to interpret the data according to strict scientific rationale

## Authors' contributions

KB and MFO conceived the idea for the study and all authors contributed to the design and concept. All authors were active in testing the questionnaire, processing the data and providing critical review of the manuscript. KB managed the testing, distribution, preparation of the data and the main responsibility for the manuscript construction. All authors interpreted the data, revised the manuscript for logical content and approved the final version.

## Pre-publication history

The pre-publication history for this paper can be accessed here:

http://www.biomedcentral.com/1472-6882/12/42/prepub

## Supplementary Material

Additional file 1 **Nationell enkät om komplementär-, integrativ och alternativmedicin till sjukvårdspersonal inom kirurgisk vård**. Click here for file
