# Supplementary material for: Knowledge about complementary, alternative and integrative medicine (CAM) among registered health care providers in Swedish surgical care: a national survey among university hospitals
Source: BMC Complement Altern Med. 2012 Apr 12;12:42. doi: 10.1186/1472-6882-12-42 (PMC3373365; doi:10.1186/1472-6882-12-42)
Supplement: Additional file 1 — Nationell enkät om komplementär-, integrativ och alternativmedicin till sjukvårdspersonal inom kirurgisk vård. [file 1472-6882-12-42-S1.DOC]

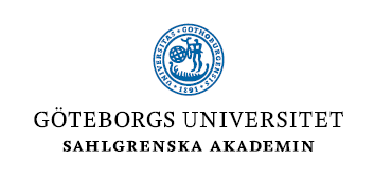


Nationell enkät om komplementär-, integrativ och alternativmedicin till sjukvårdspersonal inom kirurgisk vård

Användningen av komplementär och alternativmedicin ökar i samhället, både internationellt och i Sverige. Idag saknas kunskap kring hur personal inom hälso- och sjukvården i Sverige uppfattar detta område.

Vi har tidigare gjort en intervjustudie på detta ämne och vill nu verifiera resultatet och se om det är reproducerbart. Därför vänder sig denna enkät till Dig som är legitimerad sjukvårdspersonal och arbetar inom någon form av kirurgisk vård. Syfte är att undersöka kunskap, attityd och uppfattning kring komplementära terapier. Deltagandet i denna undersökning är frivilligt och all information som Du lämnar behandlas konfidentiellt. Alla enkäter är kodade för att påminnelse skall kunna skickas ut. Resultatet från denna enkät kommer sedan att sammanställas och publiceras i passande vetenskaplig tidskrift.

Nedan följer definitioner av medicin och terapibegrepp som berörs i denna enkät:

Konventionell = Den vård som ges inom den statliga sjukvården på sjukhus, vårdcentral eller av hemsjukvård

Komplementär = Används samtidigt som konventionell behandling, men utan att dessa anpassas efter varandra.

Alternativ = Behandling som ges istället för konventionell vård

Integrativ = Behandling som ges samtidigt som konventionell behandling och där dialog och samspel sker mellan dessa.

Kontakt vid frågor:

Kristofer Bjerså

Verksamheten för Kirurgi, Sahlgrenska Universitetssjukhuset, Göteborg

Tel. 031-3428735

E-post: [kristofer.bjersa@vgregion.se](mailto:kristofer.bjersa@vgregion.se)

Nedan finns olika behandlingsformer listade. Vilken sorts vård/terapi anser Du att de hör till? (Sätt ett kryss i ringarna för att markera Ditt svar):

|  | **Konventionell** | Komplementär | **Alternativ** | **Integrativ** | ***Känner ej till***  ***terapiform*** | **Skulle du rekommendera behandling:** |
| --- | --- | --- | --- | --- | --- | --- |
|  |  |  |  |  |  | Till patient |
| Ayurveda |  |  |  |  | ** |  JA |
| Homeopati |  |  |  |  | ** |  JA |
| Psykoterapi KBT |  |  |  |  | ** |  JA |
| Meditation, Mindfullness, etc |  |  |  |  | ** |  JA |
| Healing, Reiki, etc. |  |  |  |  | ** |  JA |
| Yoga |  |  |  |  | ** |  JA |
| Omvårdnad |  |  |  |  | ** |  JA |
| Tai Chi  Qi Gong |  |  |  |  | ** |  JA |
| Akupunktur akupressur |  |  |  |  | ** |  JA |
| Ortopedisk manuell terapi (OMT/OMI) |  |  |  |  | ** |  JA |
| Massage shiatsu  Taktilmassage |  |  |  |  | ** |  JA |
| Kiroprakti naprapati Osteopati |  |  |  |  | ** |  JA |
| Fysioterapi  SjukgymnastIK |  |  |  |  | ** |  JA |
| Örtmedicin hälsokost |  |  |  |  | ** |  JA |
| Bowen terapi |  |  |  |  | ** |  JA |
| Irisdiagnostik |  |  |  |  | ** |  JA |
| Arbetsterapi |  |  |  |  | ** |  JA |
| Kinesiologi |  |  |  |  | ** |  JA |
| Sinnesterapie t.ex. ljusterapi, musikterapi, aromterapi |  |  |  |  | ** |  JA |
| Rosenmetoden |  |  |  |  | ** |  JA |
| Zonterapi  reflexologi |  |  |  |  | ** |  JA |

KUNSKAP

|  | | | | | | | | | | | | | |
| --- | --- | --- | --- | --- | --- | --- | --- | --- | --- | --- | --- | --- | --- |
| Hur graderar Du Din kunskap inom området Komplementär-, integrativ och alternativmedicin? | | | | | | | | | | | | | |
| Ingen kunskap | | | Lite kunskap | | | | Mycket kunskap | | | | | Fullt insatt i området | |
|  | | |  | | | |  | | | | |  | |
|  | |  | |  | | |  | | | |  |  | |
| Skulle Du vilja ha mer kunskap om detta området? | | | | | | | | | | | | | |
|  JA | |  NEJ | | | |  | | |  | |  | |  |
|  | |  | | | |  | | |  | |  | |  |
| Hur viktigt anser Du det är att man som legitimerad vårdpersonal har kunskap om detta område? | | | | | | | | | | | | | |
| Helt oväsentligt | Mycket oväsentligt | | | | Ganska oväsentligt | | | Av viss vikt | | Av stor vikt | | | Av största vikt |
|  |  | | | |  | | |  | |  | | |  |
|  |  | | | |  | | |  | |  | | |  |
| Skulle Du vilja lära Dig någon eller några terapier inom detta område? | | | | | | | | | | | | |  |
|  JA |  NEJ | | | |  | | |  | |  | | |  |
|  |  | | | |  | | |  | |  | | |  |

FORSKNING

|  | | | | | |
| --- | --- | --- | --- | --- | --- |
| Hur förtrogen är Du med forskning som bedrivs inom komplementär-, integrativ- eller alternativmedicin? | | | | | |
| Aldrig hört om sådan forskning | Lite kunskap | Mycket kunskap | Fullt insatt i sådan forskning | | |
|  |  |  |  | | |
|  |  |  |  |  | |
| Skulle Du vilja ta del av resultat från forskning inom detta område? | | | | | |
|  JA |  NEJ |  | | | |
|  |  |  | | | |
| Anser Du generellt att mer forskningsresurser borde läggas på detta område? | | | | |  |
|  JA |  NEJ |  | | |  |
|  |  |  | | |  |
| Skulle Du kunna tänka Dig att delta eller driva ett forsknings- eller utvecklingsprojekt inom detta område? | | | | | |
|  JA |  NEJ |  | | | |
|  |  |  | | | |

FÖRFRÅGAN

|  | | | | | |
| --- | --- | --- | --- | --- | --- |
| Hur ofta har patienter Du träffar frågor om komplementär-, integrativ- eller alternativmedicin? | | | | | |
| Aldrig hänt | <1ggr/år | 1-2 ggr/år | 1-2 ggr/månad | 2-3ggr/veckan | >4ggr/veckan |
|  |  |  |  |  |  |
|  |  |  |  |  |  |
| Hur ofta frågar Du patienter om användning av någon form av komplementär-, integrativ- eller alternativmedicin? | | | | | |
| Aldrig hänt | <1ggr/år | 1-2 ggr/år | 1-2 ggr/månad | 2-3ggr/veckan | >4ggr/veckan |
|  |  |  |  |  |  |
|  |  |  |  |  |  |

Bakgrundsinformation

| Vilken yrkeskategori tillhör Du? |  Läkare  Sjuksköterska | | |
| --- | --- | --- | --- |
|  |  Dietist  Sjukgymnast | | |
| Hur många års erfarenhet har Du i Din profession? |  0-2 år  11-20 år | | |
|  |  3-5 år  >20 år | | |
|  |  6-10 år | | |
| Hur många års erfarenhet har Du inom kirurgisk vård? |  0-2 år  11-20 år | | |
|  |  3-5 år  >20 år | | |
|  |  6-10 år | | |
| Kön |  MAN  KVINNA | | |
| Födelse år | 19 _____ | | |
| Är Du själv utbildad i någon komplementär eller alternativ metod? |  Ja. Vilken/Vilka?:_____________________ | |  Nej |
| Utför Du någon komplementär eller alternativ metod? |  Ja, i mitt arbete |  Ja, som privatperson |  Nej |

TACK FÖR ATT DU TOG DIG TID OCH FYLLDE I ENKÄTEN!
